# Emerging Therapeutic Strategies for Heart Failure: A Comprehensive Review of Novel Pharmacological and Molecular Targets

**DOI:** 10.7759/cureus.81573

**Published:** 2025-04-01

**Authors:** Elizabeth Caroline Palaparthi, Tanvi Padala, Reva Singamaneni, Rachakatla Manaswini, Abhigna Kantula, Palle Aditya Reddy, Punuri Chandini, Addanki Sathwika Eliana, Papasani Siri Samhita, Prashanth Kumar Patnaik

**Affiliations:** 1 Department of Internal Medicine, Shasta Regional Medical Center, California, USA; 2 Department of Pharmacology, RVM Institute of Medical Sciences and Research Center, Laxmakkapally, IND

**Keywords:** artificial intelligence, crispr-cas9, heart failure, precision medicine, regenerative medicine, rna-based therapy, sglt2 inhibitors, translational research

## Abstract

Heart failure (HF) is a complex clinical syndrome characterized by the heart's inability to meet the body's metabolic demands. HF remains a global health challenge with high morbidity and mortality. Outcomes of beta-blockers, angiotensin receptor-neprilysin inhibitors (ARNIs), and mineralocorticoid receptor antagonists (MRAs) in HF remain suboptimal. HF is a heterogeneous syndrome driven by neurohormonal dysregulation, fibrosis, metabolic disturbances, and inflammation, contributing to symptoms like dyspnea, fatigue, and fluid retention.

Recent advances in pharmacological therapies, including sodium-glucose cotransporter 2 inhibitors (SGLT2 inhibitors), soluble guanylate cyclase stimulators (sGC stimulators), and cardiac myosin activators, have shown promise in HF with reduced ejection fraction (HFrEF) and HF with preserved ejection fraction (HFpEF), offering mechanism-specific interventions. Moreover, molecular-targeted therapies, such as clustered regularly interspaced short palindromic repeats (CRISPR)-associated protein 9 (Cas9) gene editing, RNA-based therapeutics, and adeno-associated virus serotype 9-sarcoplasmic reticulum calcium ATPase 2a (AAV9-SERCA2a gene) therapy, are emerging as potential disease-modifying treatments aimed at addressing genetic and inflammatory drivers of cardiomyopathies.

Artificial intelligence (AI) is transforming HF care by enhancing predictive modelling, risk stratification, and precision medicine, with applications in multi-omics data integration. AI-driven tools, including machine learning (ML) algorithms, improve echocardiographic phenotyping, optimize treatment strategies, and refine patient selection for therapies. Despite these promising developments, challenges such as data quality, standardization, scalability, and regulatory barriers remain. Furthermore, gene therapies' long-term safety and efficacy are still uncertain, with concerns about immune responses, off-target effects, and sustained gene expression. Regenerative medicine strategies, including induced pluripotent stem cells (iPSC)-derived cardiomyocytes, extracellular vesicles (EVs), and 3D-bioprinted cardiac patches, offer potential solutions for myocardial repair. However, immune rejection, graft integration, and long-term viability remain significant obstacles. Additionally, high costs associated with novel biologics and gene-based therapies limit accessibility, particularly in low-resource settings.

The future of HF management depends on overcoming these translational challenges. Key steps include validating AI-driven phenotyping tools in clinical trials, advancing scalable biomanufacturing technologies, and refining regulatory frameworks to facilitate clinical integration. By addressing these barriers, precision medicine, AI, and regenerative therapies can transform HF management, providing more personalized, effective, and accessible treatments and ultimately improving patient outcomes globally.

## Introduction and background

Heart failure (HF) is a clinical syndrome characterized by the heart's inability to pump blood effectively to meet the body's metabolic demands or to accommodate venous return at normal filling pressures. HF affects over 64 million people worldwide. HF incidences are rising due to aging populations and comorbidities like obesity, diabetes and hypertension. Low- and middle-income countries face a greater burden from infectious diseases and have limited access to advanced treatments [1]. Despite advancements in guideline-directed medical therapy (GDMT), including beta-blockers, angiotensin receptor-neprilysin inhibitors (ARNIs) and mineralocorticoid receptor antagonists (MRAs), outcomes remain suboptimal, especially for HF with preserved ejection fraction (HFpEF ≥50%) patients, where disease-modifying treatments are limited. This limited patient progress emphasizes the need for novel therapies that address HF’s underlying pathophysiology rather than just its symptoms [2].

HF is a heterogeneous syndrome driven by multiple interconnected pathophysiological mechanisms. Neurohormonal dysregulation, involving the activation of the renin-angiotensin-aldosterone system (RAAS) and sympathetic nervous system (SNS), leads to fluid retention, vasoconstriction, and maladaptive cardiac remodelling. Fibrosis results from chronic stress on the heart, disrupting myocardial function and increasing the risk of arrhythmias. Metabolic alterations, such as impaired energy production and altered substrate utilization, exacerbate myocardial dysfunction. Additionally, inflammation, marked by elevated cytokines like tumor necrosis factor-alpha (TNF-α) and interleukin-6 (IL-6), contributes to myocardial injury and endothelial dysfunction. Together, these mechanisms lead to the clinical manifestations of HF, including dyspnea, fatigue, and fluid retention, necessitating personalized therapeutic approaches [3]. Traditional therapies target neurohormonal pathways, newer pharmacological agents such as sodium-glucose cotransporter 2 (SGLT2) inhibitors, soluble guanylate cyclase (sGC) stimulators, and cardiac myosin activators offer multi-targeted interventions with significant benefits in both HF with reduced ejection fraction (HFrEF ≤40%) and HFpEF [4]. Given HFpEF’s heterogeneity, understanding of phenotype-specific strategies is essential. HFpEF is more challenging to treat than HFrEF, highlighting its complex, heterogeneous pathophysiology involving mitochondrial and endothelial dysfunction, which complicates the identification of clear therapeutic targets [5-7]. Molecular targeted therapies are redefining HF treatment by addressing genetic and inflammatory drivers [8]. Clustered regularly interspaced short palindromic repeats (CRISPR)-associated protein 9 (Cas9) gene editing, RNA-based therapeutics and adeno-associated virus serotype 9-sarcoplasmic reticulum calcium ATPase 2a (AAV9-SERCA2a) gene therapy offer potential disease-modifying solutions [9-11]. Regenerative medicine, including extracellular vesicles (EVs), 3D-bioprinted cardiac patches and induced pluripotent stem cells (iPSC)-derived cardiomyocytes, presents promising options for myocardial repair, though challenges remain in immune compatibility and functional integrations [12,13].

Artificial intelligence (AI)-driven strategies are revolutionizing HF care by enabling predictive modelling (AI-driven echocardiographic interpretation and digital biomarkers from wearable devices), risk stratification and precision medicine using multi-omics data. However, translating these innovations into clinical practice faces regulatory, scalability and ethical challenges [14]. This review examines emerging HF therapies, integrating pharmacology with molecular targeted interventions, regenerative medicine and AI-driven precision medicine to advance personalized and transformative HF management.

## Review

Data source and search strategy

A comprehensive literature search was conducted using PubMed/MEDLINE, Web of Science and Google Scholar to identify research studies on emerging HF therapies published between January 2013 and January 2025. To ensure both foundational insights and more focused on recent advancements, a structured search strategy was employed using Medical Subject Headings (MeSH) and keyword terms, focusing on HF: (“Heart failure” OR “HFrEF” OR “HFpEF”) AND (“SGLT2 inhibitors” OR “sGC stimulators” OR “cardiac myosin activators”) AND (“CRISPR-Cas9” OR “RNA-based therapeutics” OR “AAV9-SERCA2a”) AND (“extracellular vesicles” OR “iPSC-derived cardiomyocytes” OR “3D-bioprinting”) AND “AI-driven precision medicine”. Studies were screened for clinical relevance, translational significance and methodological rigor, with randomized controlled trials, meta-analyses and high-impact translational research prioritized in this review.

Selection criteria

Inclusion Criteria

This review included peer-reviewed clinical trials (near-term clinical application; phase I trials and beyond), meta-analyses, systematic reviews and translational studies on novel pharmacological agents, molecular-targeted therapies, regenerative medicine and AI-driven precision medicine for HF. Priority was given to studies on SGLT2 inhibitors, sGC stimulators, cardiac myosin activators, CRISPR-Cas9 gene editing, RNA-based therapeutics, EVs, 3D-bioprinting, iPSC-derived cardiomyocytes and AI-driven predictive models (AI-driven analytics that utilize real-world data to inform clinical decision-making). Research incorporating multi-omics data, ML applications, or real-world evidence (RWE; specifically considered retrospective cohort studies) was also included.

Exclusion Criteria

Studies focusing solely on conventional HF management, case reports, commentaries, conference abstracts and non-peer reviewed sources were excluded. Small sample studies with unclear endpoints or insufficient translational relevance were omitted. Preclinical studies without near-term clinical application were also excluded. The final database was carefully reviewed using the following sections.

Mechanisms and efficacy of novel pharmacological agents in HF sub-types

HF is a complex syndrome and is grossly classified into HFrEF and HFpEF, each requiring targeted therapeutic approaches [15]. While traditional therapies address specifically neurohormonal dysregulation, emerging pharmacological agents offer mechanism-specific interventions, addressing metabolic, contractile, and vascular abnormalities [4].

SGLT2 Inhibitors: Metabolic Modulation and Cardio-Protection

SGLT2 was originally developed for diabetes, exerting beneficial effects beyond metabolic modulation, including diuretic-like effects, reduction in oxidative stress, and favorable impacts on adipokine profiles, all of which contribute to improved cardiovascular outcomes [16]. Trials like Dapagliflozin and Prevention of Adverse Outcomes in Heart Failure (DAPA-HF) and Empagliflozin Outcome Trial in Patients with Chronic Heart Failure and a Reduced Ejection Fraction (EMPEROR-Reduced) demonstrated significant reductions in HF hospitalizations and mortality in HFrEF patients [17,18]. In HFpEF, EMPEROR-Preserved and Dapagliflozin Evaluation to Improve the lives of Patients with Preserved Ejection Fraction Heart Failure (DELIVER) trials reported improved exercise capacity and symptom relief, particularly in HF patients with metabolic dysfunction [17,18].

Cardiac Myosin Activators: Enhancing Systolic Function

Unlike traditional inotropes, cardiac myosin activators (e.g., omecamtiv mecarbil, aficamten) enhance sarcomere contractility (Omecamtiv mecarbil, unlike traditional inotropes, does not increase intracellular calcium. Instead, it enhances sarcomere contractility by increasing the time of systolic ejection without raising myocardial oxygen demand, thus avoiding the arrhythmic risks commonly associated with traditional inotropic therapies). By prolonging systolic ejection time, they improve stroke volume and cardiac output, reducing ventricular workload. The Global Approach to Lowering Adverse Cardiac Outcomes Through Improving Contractility in Heart Failure (GALACTIC-HF) trial showed a modest reduction in HF hospitalizations, particularly in patients with severely reduced ejection fraction (≤30%) [19]. Aficamten, a next-generation myosin inhibitor, is being explored for hypertrophic cardiomyopathy (HCM) and hypercontractile HFpEF sub-types, but limited clinical data is available [20].

sGC Stimulators: Targeting Vascular Dysfunction

HFpEF is often driven by vascular dysfunction, leading to pulmonary congestion and systemic hypertension. sGC stimulators (e.g., vericiguat) restore nitric oxide (NO)-soluble guanylate cyclase-cyclic guanosine monophosphate pathway (NO-sGC-cGMP) signalling and improve vascular stiffness and myocardial relaxation. The Vericiguat Global Study in Subjects With Heart Failure With Reduced Ejection Fraction (VICTORIA) trial demonstrated a 15% reduction in HF hospitalizations in high-risk HFrEF patients, supporting vericiguat’s use as an adjunct to GDMT. Praliciguat, a dual sGC stimulator/PDE5 inhibitor, is under investigation for HFpEF with pulmonary hypertension, with preliminary data showing improved pulmonary resistance and right ventricular function. Further, the VICTORIA trial specifically studied sGC stimulators in HFrEF patients, and as such, the proven efficacy of sGC stimulators in HFpEF remains investigational. Thus, ongoing research is evident on the evolving landscape of sGC stimulator therapy in HFpEF [21,22].

Integrating Novel Agents Into HF Precision Medicine

Clinical trials highlight the potential benefits of precision medicine approaches, SGLT2 inhibitors combined with sGC stimulators for vascular dysfunction in HFpEF and myosin activators with ARNIs for contractile dysfunction in HFrEF and emerging clinical evidences are currently under investigation. For instance, the combination of SGLT2 inhibitors (e.g., empagliflozin) with sGC stimulators (e.g., vericiguat) in HFpEF is being explored in ongoing trials such as the VITALITY-HFpEF trial, which aims to assess their combined effect on improving vascular function and reducing hospitalization in HFpEF patients [17,18]. Some studies emphasize the clinical utility of echocardiographic parameters for managing end-stage HF (e.g., E/e' SR as a reliable predictor of short-term mortality of patients with HFrEF) [23]. Multi-omics and AI-driven stratification refine HF phenotyping therapies that may become highly individualized, transforming HF management into a personalized, mechanism-specific approach. ML algorithms are increasingly being used for echocardiographic phenotyping, where AI-driven models can analyze echocardiographic images to identify distinct HF subtypes and predict treatment responses [14].

Molecular-targeted therapies for genetic and inflammatory HF sub-types

Emerging molecular therapies, including CRISPR-Cas9 gene editing, RNA-based therapeutics, AAV9-SERCA2a gene therapy and cytokine-targeting biologics, offer precision medicine approaches to correct genetic abnormalities, modulate inflammation and restore cardiomyocyte function, advancing disease-modifying HF treatments [9,24].

Genetic mutations contribute to 30-40% of dilated cardiomyopathy (DCM) and HCM cases, with titin truncating variant (TTNtv), myosin-binding protein C3 (MYBPC3), lamin A/C gene (LMNA) and BCL2-associated athanogene-3 (BAG3) among the most common pathogenic variants. These mutations disrupt sarcomere integrity, leading to progressive HF. CRISPR-Cas9 gene editing offers the potential to correct or silence these mutations, restoring normal function. Preclinical studies have successfully corrected TTNtv mutations in iPSC-derived cardiomyocytes, improving sarcomere function. Similarly, MYBPC3 mutations in HCM have been edited, reducing myocardial hypertrophy. However, off-target effects, immune responses to viral vectors and in vivo delivery challenges remain significant hurdles. To enhance safety, base editing and prime editing techniques allow more precise single-nucleotide corrections with minimal DNA damage. Beyond editing, AAV9-SERCA2a gene therapy targets calcium homeostasis, a critical dysfunction in HF. SERCA2a downregulation impairs calcium cycling, leading to myocardial dysfunction. While Calcium Upregulation by Percutaneous Administration of Gene Therapy in Cardiac Disease (CUPID-1) demonstrated improved ventricular function with AAV9-SERCA2a overexpression, the CUPID-2 trial failed to replicate these benefits due to poor vector transduction [9,24].

RNA-Based Therapeutics for Myocardial Remodelling and Metabolism

RNA-based interventions, such as antisense oligonucleotides (ASOs) and small interfering RNAs (siRNAs), provide an alternative strategy for HF gene modulation. MicroRNA-132 (miR-132), a key regulator of maladaptive remodelling, is identified as a target for intervention. Cardior-132-L (CDR132L) is currently in Phase 1 clinical trials, focusing on safety and efficacy in reducing myocardial fibrosis and improving heart function, with endpoints including changes in left ventricular function and fibrosis biomarkers. Another promising RNA therapy is inclisiran, which is known for its lipid-lowering effects. It may also benefit HFpEF patients by improving endothelial function and reducing inflammation, in addition to atherosclerosis reduction, particularly in HFpEF patients with systemic inflammation and dyslipidemia [25-27].

Targeting Inflammation in HFpEF With Biologics

Chronic inflammation plays a major role in HFpEF, with elevated IL-6, TNF-α, and C-reactive protein (CRP) levels correlating with worse outcomes. Targeting inflammation in HFpEF with biologics like ziltivekimab shows potential beyond biomarker reduction. In trials, it reduced hs-CRP and improved vascular function and exercise capacity, which may help reduce hospitalizations, though long-term mortality data is limited. Given HFpEF's heterogeneity, combining anti-inflammatory biologics with metabolic or vascular-targeting therapies could be more effective in addressing the diverse mechanisms of the disease [28]. Given the increasing recognition of inflammation-driven HF subtypes, biosimilars and alternative delivery methods, such as lipid nanoparticles (LNPs) or exosome-based carriers, could help reduce costs and improve the accessibility of biologic therapies for HFpEF [28,29].

Thus, molecular-targeted therapies show promise, but they are still in the investigational stages and far from routine clinical use. Ethical concerns, including off-target mutations and potential germline modifications, remain significant challenges. AI could refine patient selection for gene/RNA-based therapies, such as using polygenic risk scores or transcriptomic profiling to identify patients most likely to benefit from specific treatments, thereby personalizing therapy and improving outcomes.

Regenerative and tissue-engineering strategies for myocardial repair

HF following myocardial infarction (MI) remains a major challenge due to the limited regenerative capacity of adult cardiomyocytes. While current pharmacological and device-based therapies alleviate symptoms, they do not restore lost myocardial tissue. Regenerative strategies, including engineered EVs, 3D-bioprinted cardiac patches and iPSC-derived cardiomyocytes, are emerging as promising solutions for myocardial repair. However, challenges related to immune rejection, graft integration and long-term viability remain key obstacles to clinical translation [30,31].

Engineered EVs for Myocardial Repair

EVs, particularly exosomes from mesenchymal stem cells (MSCs) and cardiac progenitor cells, act as cell-free therapeutic agents that deliver cardioprotective micro RNAs, growth factors and anti-apoptotic molecules to injured myocardium. Engineered EVs enriched with miR-21, miR-126, and vascular endothelial growth factor (VEGF) have shown enhanced neovascularization, fibrosis reduction and improved cardiac function in preclinical models. Strategies such as surface modification for targeted homing and RNA-enhanced EVs are being explored to optimize regenerative efficacy [30,31].

Tissue-Engineered Cardiac Patches and 3D Bio-Printing

3D-bioprinted cardiac patches, composed of biocompatible scaffolds with cardiomyocytes, endothelial cells and fibroblasts, provide structural and electrical support while facilitating cellular integration and vascularization. Preclinical studies show that bioengineered patches improve contractility, prevent adverse remodelling and enhance post-MI survival. Hydrogel-based scaffolds incorporating angiogenic factors (VEGF and fibroblast growth factor, or FGF) and conductive materials (graphene, gold nanoparticles) are being developed to enhance vascularization and electrical coupling with host tissue [32].

iPSC-Derived Cardiomyocytes for Cell-Based Therapy

iPSCs offer an unlimited autologous source of cardiomyocytes for transplantation. These cells can electrically integrate into the host myocardium, restoring function. However, challenges such as arrhythmias (incomplete ion channel expression and abnormal action potentials), poor survival, low differentiation efficiency and immune rejection hinder clinical translation. Advances in CRISPR gene editing are generating hypoimmunogenic iPSC-derived cardiomyocytes, reducing the need for immunosuppression. Additionally, other stem cell sources, like cardiac progenitor cells and MSCs, are being explored as alternatives due to their potentially more mature and less arrhythmogenic characteristics [33].

Key challenges for myocardial regeneration include immune rejection, graft integration and scalability. Immune compatibility and functional integration remain significant hurdles for regenerative therapies, particularly for cell-based interventions like iPSC-derived cardiomyocytes. Furthermore, the scalability of these therapies is also a concern, as the manufacturing of sufficient quantities of cells or EVs for widespread clinical use is complex and costly. Recent developments, such as optimizing culture conditions and utilizing immune-evasive iPSCs, aim to address immune rejection and improve engraftment success. However, while some promising preclinical studies show functional integration, the long-term durability and engraftment remain unclear, with the risk of arrhythmias and insufficient cellular survival over time [30-33].

Applications of AI

AI is transforming HF management by enabling predictive analytics, precision medicine and real-time monitoring. AI-driven models are enhancing diagnosis, risk stratification, treatment optimization and drug discovery, offering a data-driven approach to personalized care [14].

AI-Driven Predictive Modelling and Risk Stratification

ML algorithms are improving HF risk prediction by analyzing electronic health records (EHRs), multi-omics data and wearable sensor inputs. AI-based models have outperformed traditional risk scores in predicting HF decompensation, hospitalization and mortality. For example, deep learning algorithms, including convolutional neural networks (CNNs) and recurrent neural networks (RNNs), have been applied to EHRs data and imaging modalities (e.g., cardiac magnetic resonance imaging (MRI) and echocardiograms) to predict hospitalizations, readmissions, and mortality in HF patients with higher precision than traditional models. Additionally, the concept of digital twins creating patient-specific simulations to predict disease progression and response to therapy is gaining traction with recent studies exploring its use in HF management [34].

AI-Enhanced Imaging and Phenotyping

Deep learning algorithms are revolutionizing cardiac imaging, improving the automated assessment of echocardiograms, cardiac MRI and CT scans. AI-assisted imaging can quantify left ventricular function, myocardial fibrosis and diastolic dysfunction with higher accuracy than manual interpretation. Additionally, ML-based HFpEF phenotyping models integrate clinical, biomarker and imaging data to classify patients into pathophysiologically distinct subgroups, facilitating targeted therapies. However, their validation across diverse imaging (e.g., echocardiogram) datasets and healthcare systems is still a work in progress [35].

AI in Precision Medicine and Drug Discovery

AI is advancing precision medicine by integrating genomic, proteomic and metabolic profiles to identify HF phenotypes that respond to specific therapies. AI-driven analyses of SGLT2 inhibitor and sGC stimulator trials have identified HF subgroups with superior clinical benefits. Moreover, AI is accelerating drug discovery by analyzing biological datasets, predicting drug-target interactions and repurposing existing drugs for HF treatment. As AI continues to evolve, its integration with digital health technologies, RWE and adaptive clinical trials will optimize HF management and treatment selection. AI models such as deep learning, random forests, and reinforcement learning have shown effectiveness in predicting HF outcomes, adoption is still growing. Efforts are underway to create personalized risk stratification models that tailor predictions to individual patient phenotypes, incorporating clinical, genetic, and other personalized data to improve accuracy and outcomes. However, challenges related to data bias, interpretability and ethical considerations must be addressed to ensure equitable and clinically relevant applications [34,35].

Pathophysiological complexity of HFpEF through precision medicine

HFpEF accounts for nearly half of all HF cases and presents substantial heterogeneity, making broad-spectrum treatments ineffective. Unlike HFrEF, HFpEF involves systemic inflammation, endothelial dysfunction, metabolic disturbances and myocardial fibrosis, necessitating precision medicine approaches using multi-omics, biomarker-driven phenotyping and AI-based therapy selection to target distinct sub-phenotypes [36].

HFpEF is now seen as a collection of phenotypically distinct conditions rather than a single disease. Proteomics, metabolomics, and transcriptomics have refined patient classification with ML models, such as those from the Heart Failure Molecular Epidemiology for Therapeutic Targets (HERMES-HF) consortium, identifying HFpEF subgroups with varying therapeutic responses. Inflammatory HFpEF, often linked to obesity and chronic kidney disease, is driven by IL-6, TNF-α and CRP elevation, contributing to vascular inflammation and stiffness. IL-6 inhibitors (ziltivekimab) and SGLT2 inhibitors are being investigated for their cardioprotective effects. Metabolic HFpEF, prevalent in diabetes and obesity, is characterized by insulin resistance and mitochondrial dysfunction. SGLT2 inhibitors and GLP-1 receptor agonists (semaglutide) have shown promise in improving myocardial energetics, reducing inflammation and promoting weight loss. Fibrotic HFpEF is marked by excessive collagen deposition and TGF-β activation, leading to diastolic dysfunction. Antifibrotic agents (pirfenidone, MRAs) are being explored for reversing fibrosis. Vascular dysfunction HFpEF, involving microvascular dysfunction and impaired nitric oxide (NO) signaling, responds to sGC stimulators (vericiguat, praliciguat), particularly in patients with pulmonary hypertension [36,37].

AI-driven models are refining HFpEF risk prediction, subphenotyping and therapy selection and enhancing diagnostic accuracy using biomarker integration, echocardiography and cardiac MRI. ML has optimized drug selection, identifying patients most responsive to SGLT2 inhibitors and sGC stimulators. Precision medicine is now shaping HFpEF treatment algorithms, incorporating biomarker-driven approaches such as mitochondrial-targeted peptides (elamipretide), vascular stabilizers (sulodexide, SGLT2 inhibitors) and anti-inflammatory agents (IL-6 inhibitors, omega-3 fatty acids, antifibrotics) [34,36-38].

HF management is shifting from empirical treatments to precision-based interventions driven by multi-omics, AI-driven phenotyping and RWE. Their prospective validation in interventional trials is still limited. Barriers to real-world implementation, such as the cost and accessibility of multi-omics data, as well as reproducibility issues, pose challenges for clinical translation. However, AI is increasingly overcoming data quality and standardization issues, particularly through natural language processing and data imputation techniques. AI-assisted treatment decisions, though still in early stages, are beginning to show improvements in real-world outcomes. Efforts to train clinicians, integrate AI insights into clinical guidelines, and address racial and sex-based disparities in AI-driven decision-making are crucial for ensuring fairness and maximizing the impact of AI in HF care [14,34,35,38].

Translational challenges and ethical implications of emerging therapies

The integration of novel pharmacological agents, molecular therapies and regenerative medicine into HF management faces significant translational challenges and ethical concerns. Despite their promise, clinical implementation must overcome hurdles related to scalability, long-term safety, regulatory approval, cost-effectiveness and equitable access [39].

Scientific and Logistical Challenges

Gene-editing technologies like CRISPR-Cas9 carry risks of off-target mutations, immune reactions and uncertain long-term effects. Similarly, RNA-based therapies require optimized delivery systems (e.g., LNPs or exosome-based transport) to enhance stability and target specificity. Immune rejection remains a significant challenge for iPSC-derived cardiomyocyte therapies. Researchers are tackling this issue by developing hypoimmunogenic iPSCs, which are genetically modified to express reduced levels of major histocompatibility complex (MHC) antigens, making them less recognizable by the immune system. Additionally, novel immune-evasive strategies, such as human leukocyte antigen (HLA)-knockout cells, are being explored to create iPSCs that are less prone to immune recognition, thus reducing the risk of rejection and the need for immunosuppression. RNA-based and gene therapies face significant challenges in delivery efficiency and targeting. LNPs and viral vectors are at the forefront of these efforts. LNPs are being optimized to enhance their stability, cellular uptake, and tissue targeting. For gene therapies, self-complementary adeno-associated viruses (scAAVs) and cardiac-specific promoters are being developed to improve transduction efficiency and direct the therapeutic gene expression specifically to cardiac tissue, thus increasing the effectiveness of the therapy. These innovations aim to improve the precision and efficacy of RNA and gene delivery, particularly for cardiac applications [39,40].

Regulatory and Economic Barriers

Stringent regulatory processes demand extensive long-term safety data for gene and cell-based therapies. The high costs of biologics, regenerative therapies and AI-driven precision medicine raise concerns about affordability, especially in low and middle-income countries. Biosimilars are helping reduce the cost of cardiac drugs, making essential therapies more accessible, while AI is significantly lowering drug development costs through virtual screening, adaptive trial designs, and optimized patient selection algorithms. Industry efforts to automate iPSC production are improving scalability and reducing manufacturing costs, which is crucial for the widespread use of cell-based therapies. Additionally, AI is streamlining regulatory approval processes by enhancing clinical trial analytics and providing real-time data insights, which accelerates the approval of gene therapies and regenerative medicine. These innovations are collectively driving progress in making advanced therapies more efficient, affordable and accessible [41].

Ethical and Social Considerations

Ethical issues, particularly with gene editing and germline modifications, necessitate rigorous oversight and clear regulatory frameworks. High-cost therapies risk widening healthcare disparities, emphasizing the need for equitable access and thorough informed consent. Algorithmic bias in AI-driven HF management can lead to disparities in treatment outcomes across demographic groups, with models often underperforming for minority populations due to unrepresentative training data. To mitigate this, efforts are being made to diversify datasets and improve fairness. Ethical concerns about AI in HF treatment include the lack of transparency and explainability, raising issues around patient autonomy and accountability. Efforts are ongoing to develop explainable AI to enhance trust in AI-driven decision-making. On the regulatory front, global initiatives are focusing on ensuring the affordability of advanced therapies through biosimilar approval processes and government-backed strategies, aiming to improve access to gene and cell-based HF treatments, especially in LMICs [40-42].

Challenges and future directions

Despite significant advancements in novel pharmacological agents, molecular-targeted therapies, regenerative medicine and AI-driven precision medicine, several challenges hinder their seamless integration into routine HF management. Overcoming these barriers is essential to ensure safety, scalability, affordability and clinical applicability.

Long-term safety and efficacy remain a primary concern. Gene-editing technologies like CRISPR-Cas9 pose risks of off-target mutations, immune reactions and unintended genomic alterations. Similarly, RNA-based therapies and AAV-mediated gene delivery require extensive long-term monitoring to assess durability and potential adverse effects. In regenerative medicine, challenges such as poor engraftment, limited cell survival and arrhythmogenic risks must be addressed before widespread clinical adoption of iPSC-derived cardiomyocytes and engineered EVs. Regulatory and economic barriers further complicate the adoption of emerging HF therapies. Approval pathways for gene and cell-based treatments demand rigorous long-term safety data and adaptive trial designs, prolonging regulatory timelines. Additionally, high costs of biologics, precision medicine and regenerative therapies pose accessibility challenges, particularly in low-resource settings. Strategies such as biosimilar development, automated iPSC production and scalable biomanufacturing solutions are needed to reduce production costs and improve affordability.

Overcoming current challenges in HF management requires leveraging innovative solutions such as RWE and AI-driven platforms. RWE accelerates therapy validation and regulatory approvals, while AI significantly enhances patient stratification, precision medicine, and response prediction for gene or RNA-based therapies. Moving forward, successful adoption of AI-driven HF management depends on prospective validation, integration into clinical practice, clinician training, and rigorous regulatory frameworks ensuring fairness and transparency. These strategies collectively promise more personalized, accessible, and effective HF care.

## Conclusions

Emerging therapies in HF demonstrate clear phenotype-specific benefits, with SGLT2 inhibitors effectively improving outcomes across both HFpEF and HFrEF and sGC stimulators like vericiguat significantly benefiting worsening HFrEF patients. Although promising, CRISPR-Cas9 gene editing, RNA-based therapeutics, and gene therapies face substantial translational hurdles, including immune responses, off-target genetic effects, and challenges in achieving controlled, durable gene expression issues actively addressed by novel strategies like immune-evasive cells and advanced viral delivery systems. AI is notably advancing HF care through practical applications such as EHR-integrated risk prediction tools and clinical decision support systems, though disparities in data quality and ethical considerations around bias and data privacy remain critical. High costs and scalability limitations, exemplified by complex biomanufacturing processes for iPSC-derived cardiomyocytes and the economic burden of gene therapies, continue to restrict widespread clinical adoption.

Future research must emphasize the prospective validation of AI-driven phenotyping, the development of scalable production technologies, refinement of ethical and regulatory frameworks, and integration of multi-omics-based precision medicine to ensure these advancements translate effectively into personalized and equitable HF management.
